# Tuberculosis: tracheal involvement

**DOI:** 10.1590/0100-3984.2015.0200

**Published:** 2016

**Authors:** Brainner Campos Barbosa, Viviane Brandão Amorim, Luiz Flávio Maia Ribeiro, Edson Marchiori

**Affiliations:** 1 Hospital Samaritano - Clínica Luiz Felippe Mattoso, Rio de Janeiro, RJ, Brazil.; 2 Clínica Felippe Mattoso, Rio de Janeiro, RJ, Brazil.; 3 Universidade Federal do Rio de Janeiro (UFRJ), Rio de Janeiro, RJ, Brazil.

**Figure 1:**
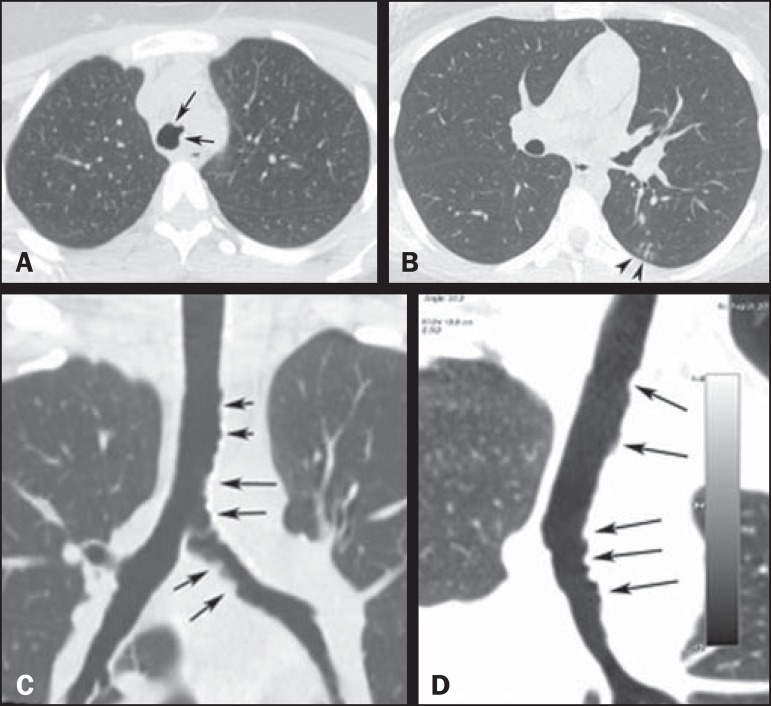
**A:** Axial CT slice showing irregular narrowing of the tracheal lumen (arrows). **B:** Axial CT slice showing centrilobular opacities, with a tree-in-bud aspect, in the lower lobe of the left lung, suggesting bronchiolar filling. **C,D:** Coronal and oblique coronal reconstructions showing irregular internal contours, together with parietal thickening (arrows), in the trachea and the left main bronchus.

Dear Editor,

A previously healthy 22-year-old female sought medical attention, complaining of productive cough and hoarseness. She reported no other respiratory or constitutional symptoms. Physical examination revealed discrete stridor. For diagnostic clarification, computed tomography (CT) of the chest was performed The CT scan showed grouped, branching centrilobular opacities, with the "tree-in-bud" aspect, suggesting distal bronchiolar filling. The trachea and left main bronchus presented irregular internal contours, with nodular thickening of the walls (Figure 1), together with a discrete increase in the density of the mediastinal fat adjacent to those changes. Sputum examination was conducted and was positive for tuberculosis, confirming the clinical and radiological suspicion of tracheobronchial tuberculosis. Specific treatment was started and resulted in resolution of the findings.

In patients with tuberculosis, tracheal involvement is relatively uncommon, occurring in only 4% of those with the endobronchial form of the disease^(1-3)^. Tracheobronchial tuberculosis mainly affects younger, female patients, its incidence peaking in the third decade of life. The disease can affect the greater part of the trachea, also affecting the bronchi, or it can affect just a small segment of the trachea or of one bronchus^(4,5)^. The clinical presentation can be insidious, simulating bronchogenic carcinoma, or acute, with a profile similar to that of asthma, foreign body aspiration, or pneumonia. In most cases, patients with tracheobronchial tuberculosis present a productive cough, hemoptysis, chest pain, generalized weakness, fever, dyspnea and bronchorrhea^(1,3)^. In cases that are more severe, there can be acute tracheal obstruction^(6)^. The main complications are fibrotic scarring and tracheobronchial stenosis, an accurate diagnosis and early treatment being crucial^(6)^.

The differential diagnoses include other diseases affecting the trachea, not only those presenting localized involvement- such as primary tracheal neoplasms, injuries of traumatic origin, and some infectious diseases-but also those presenting diffuse involvement-amyloidosis, tracheobronchopathia osteochondroplastica, relapsing polychondritis, laryngotracheobronchial papillomatosis, tracheobronchomegaly, neurofibromatosis, Wegener's granulomatosis, lymphoma, and paracoccidioidomycosis^(5,7-12)^.

Imaging studies have become increasingly important in the evaluation of chest diseases, as recently noted in the radiology literature of Brazil^(13-19)^. In the study of the trachea, imaging studies comprise X-rays and, primarily, CT of the chest, which can show irregular, circumferential narrowing of the lumen, with or without mediastinitis. In fibrotic disease, the lumen is smoother and the wall is not thickened. Lymphadenopathy is generally associated with active tuberculosis^(4,6)^.

Bronchoscopy can reveal inflamed mucosa, submucosal granuloma or polyp, ulceration, hypertrophy, or cicatricial stenosis; histologically, tracheobronchial tuberculosis can be identified the presence of giant cell granuloma and caseous necrosis^(1)^. Although the gold standard for the diagnosis of tracheobronchial tuberculosis is the finding of granulomas in the tracheal/bronchial mucosa, a diagnosis based on imaging findings and sputum positivity is accepted and enables immediate treatment^(2)^.

Making a diagnosis of tracheobronchial tuberculosis requires suspicion, and it is necessary to correlate the clinical manifestations with the radiological findings. Early diagnosis and treatment can avert the complications of the disease.
